# Combination of arbuscular mycorrhizal fungi and phosphate solubilizing bacteria on growth and production of *Helianthus tuberosus* under field condition

**DOI:** 10.1038/s41598-021-86042-3

**Published:** 2021-03-22

**Authors:** Sabaiporn Nacoon, Sanun Jogloy, Nuntavun Riddech, Wiyada Mongkolthanaruk, Jindarat Ekprasert, Julia Cooper, Sophon Boonlue

**Affiliations:** 1 Department of Microbiology, Faculty of Science, Khon Kaen University, Khon Kaen, 40002 Thailand; 2 Department of Agronomy, Faculty of Agriculture, Khon Kaen University, Khon Kaen, 40002 Thailand; 3 School of Natural and Environmental Sciences, Agriculture Building, Newcastle University, Newcastle Upon Tyne, NE1 7RU UK

**Keywords:** Bacteria, Microbial communities, Fungi, Arbuscular mycorrhiza

## Abstract

In this work, the effects of co-inoculation between an arbuscular mycorrhizal fungus (AMF) and a phosphate solubilizing bacteria (PSB) to promote the growth and production of sunchoke under field condition were investigated during 2016 and 2017. Four treatments were set up as follows: plants without inoculation, with AMF inoculation, with PSB inoculation and with co-inoculation of PSB and AMF. The results showed the presence of PSB and AMF colonization at the harvest stage in both years. This suggested the survival of PSB and successful AMF colonization throughout the experiments. According to correlation analysis, PSB positively affected AMF spore density and colonization rate. Also, both AMF and PSB positively correlated with growth and production of sunchoke. Co-inoculation could enhance various plant parameters. However, better results in 2016 were found in co-inoculation treatment, while AMF inoculation performed the best in 2017. All of these results suggested that our AMF and PSB could effectively promote growth and production of sunchoke under field conditions. Such effects were varied due to different environmental conditions each year. Note that this is the first study showing successful co-inoculation of AMF and PSB for promoting growth and yield of sunchoke in the real cultivation fields.

## Introduction

Jerusalem artichoke or sunchoke (*Helianthus tuberosus* L.) originated in North America and is now widely cultivated in temperate areas around the northern and southern hemispheres. The crop does not usually grow well in tropical areas, especially in humid lowlands^1^. Sunchoke tubers are a rich source of inulin, a fructose polymer used as additives in healthy foods and industrial products such as diabetic foods, inulin-supplemented prebiotics and animal feeds^2^. Inulin and its degradation products are known as prebiotics, which are capable of stimulating growth of gut microbiota in the human colons^3^. Sunchoke has also been used to produce biofuels and used as a dietary fibre in food manufacturing^4^. Due to its wide variety of applications, sunchoke has become one of the most economical crops in many areas. In order to increase yield of sunchoke, chemical fertilizers have been usually applied in the fields. Although the use of chemical fertilizers can rapidly increase plant growth and yield, it can cause nutrient imbalance in soils^5,6^. Therefore, recent research has shed a light on finding the more environmentally friendly ways to improve plant growth and yield. One of those alternative approaches is the use of plant-associated microorganisms which can exchange nutrients with plants such as plant growth promoting microorganisms (PGPM). PGPM receive nutrients from plants while colonizing the rhizosphere or within plant cells, and in return promoting plant growth through solubilizing nutrients, producing phytohormones and inducing plant immune responses^7^. Among various groups of PGPM, arbuscular mycorrhizal fungi (AMF) and phosphate solubilizing bacteria (PSB) are the most commonly studied. PSB are microbes that can solubilize phosphorus in the soil resulting in enhanced available phosphorus (P) for plants. Moreover, to enhance plant growth, PSB can also provide plant hormones such as indole acetic acid (IAA)^8^, cytokinin^9^ and gibberellins^10^, releasing siderophores and producing hydrogen cyanide^11^ and 1-aminocyclopropane-1-carboxylic acid (ACC) deaminase^12^. Due to those potentialities, PSB have been widely used as inoculants to increase P uptake and crop yield. Similarly, AMF can increase plant nutrient uptake^13^, facilitate plant tolerance to soil pathogens^14^ and improve plant resistance to abiotic stresses such as drought^15^ and metal pollution in soils^16^. In addition, AMF colonization in plant roots can help to balance hydration in plant cells^17^. Due to different functions of PSB and AMF, it is interesting to investigate synergistic effects of these 2 types of PGPM to enhance plant growth and yield.

Co-inoculation of AMF and PSB for promoting plant growth is presumably more beneficial than single inoculation, in which AMF living within plant cells help plants uptake soluble P released by PSB living outside plant roots. Previous research reported that co-inoculation of AMF and PSB can result in enhanced P uptake in maize under greenhouse conditions^18^. More evidence on the co-inoculation of AMF and PSB has been reported with many types of plants including *Solanum lycopersicum* L.^19^, *Eleusine coracana*^20^, linseed^21^, wheat^22^, carrot and potato^23^ and broad beans^24^. This suggested that the co-inoculation of PSB and AMF is more beneficial to plants than a single inoculation of either one of them. Our previous work^25^ under pot trial conditions showed that a dual culture between an AMF strain *Rhizophagus intraradices* KKU-Wh and a PSB strain *Klebsiella variicola* KKU-UDJA102x89-9 significantly increased plant growth parameters of sunchoke. However, the study on the interaction of these 2 types of organisms in enhancing growth and production of sunchoke under field conditions is still limited. We hypothesized that the use of PSB and AMF inoculums is likely to have a positive effect on enhancing available phosphorus providing a healthy environment for AMF to function, and thus improving plant growth and productivity. This work then focused on the synergistic effects of PSB and AMF on stimulating growth of sunchoke under field conditions in 2 experimental years (2016 and 2017). This investigation would offer an opportunity to develop biological inoculants from the co-culture of AMF and PSB for increasing the growth and production of sunchoke under field conditions in the future.

## Results

The field soil used for this experiment was sand in both years. Chemical compositions and physiological properties of the field soil were shown in Table 1. The results indicated that soil properties in 2016 were different from those of 2017. Considering physical properties, soil in 2016 was composed of a lower amount of clay (1.16%) than that of in 2017 (4.00%). However, both soils 2016 and 2017, containing a relatively equal amount of sand (~ 89%) and silt (~ 7–9%), were classified as sand. Moreover, soil in 2017 contained a lower amount of nutrients (Nitrogen (N), Phosphorus (P) and Potassium (K)) than that of soil in 2016. Soils in both years had a relatively equal amount of organic matter (~ 0.4–0.5%) and pH (~ 7). These results suggested that 2016 was likely more fertile than soil in 2017.

**Table 1:** Chemical compositions and physical properties of the field soil used for field experiments in 2016 and 2017.

Soil properties	Experimental year	
	2016	2017
**Physical properties**		
Sand (%)	89.00	88.90
Silt (%)	9.86	7.00
Clay (%)	1.16	4.00
Texture class	Sand	Sand
**Chemical properties**		
pH (1:1 H_2_O)	6.53	6.70
Organic matter (%)	0.50	0.40
Total N (mg kg^−1^ soil)	249.24	200.00
Available P (mg kg^−1^ soil)	47.51	37.70
Exchangeable K (mg kg^−1^ soil)	35.27	30.10

**Figure 1:**
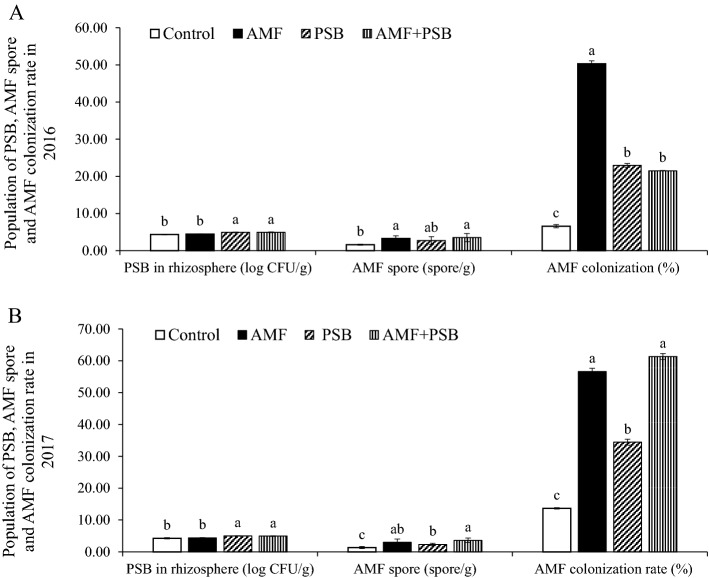
(**A**) The PSB population, the amount of AMF spores in rhizosphere soil and the percentage of root colonization at the harvest stage in 2016 and (**B**) in 2017. Mean values with the same letters are not significantly different among treatments of the same parameter at *P* ≤ 0.05 when compared by LSD. Means are the average values of the data from 12 replications.

Figure 1 presented the PSB population, the amount of AMF spores in rhizosphere soil and %root colonization of AMF at the harvest stage in 2016 (Fig. 1A) and 2017 (Fig. 1B). The results showed that the PSB population in rhizosphere soil in the PSB and AMF + PSB treatments were significantly higher than that of the other treatments in both experimental years. Also, the number of AMF spores in all inoculated treatments were higher than that of the uninoculated controls in both years. This resulted in higher root colonization in all inoculated treatments than that of the uninoculated ones. All of these results suggested successful AMF colonization in plant roots and PSB survival in rhizosphere soils throughout the experiment.

Plant growth parameters including SPAD value, plant height, leaf area index (LAI), inulin content and nutrient uptake (N, P, K) of sunchoke grown under different treatments in 2016 and 2017 were presented in Table 2. The results showed that the SPAD value of the inoculated plants was not significantly different to that of the uninoculated controls in both years 2016 and 2017. In 2016, a significant increase in LAI and K uptake by plants was found in all inoculated plants, whereas no significant difference in N and P uptake was observed when compared to the uninoculated controls. However, P content in plants inoculated with PSB alone seemed to be higher than the others. This suggested the presence of phosphate solubilization activity of the PSB. It was found that the inoculation of PSB alone positively affected only LAI and K uptake by plants, while the inoculation of AMF alone could also increase the inulin content up to ~ 42%, which was twice as much of that of the controls. Similarly, the AMF + PSB co-inoculated plants had significantly higher plant height, LAI, and inulin content than those of the uninoculated controls. These results in 2016 suggested that co-inoculation of AMF and PSB could better enhance the growth of sunchoke than single inoculations and non-inoculation. Contrarily, in 2017, AMF was likely able to play a more important role in promoting plant growth than PSB and co-inoculation between AMF and PSB. Plants inoculated with AMF had significantly higher values in almost all parameters, except SPAD value and P uptake than those of the other treatments, while PSB could increase LAI, inulin content and P uptake. This evidence that an increase in P content (and not other environmental factors) in PSB-treated plants affected directly PSB. It was found that co-inoculation of AMF and PSB could significantly enhance plant height, LAI and inulin content, which were relatively similar to the results in 2016. This suggested that co-inoculation of AMF and PSB mainly affected those 3 parameters.

**Table 2:** Effect of PSB and AMF inoculation on SPAD value, height, leaf area index (LAI), inulin content and nutrient concentrations of sunchoke evaluated at the harvest stage in 2016 and 2017.

Treatments	SPAD value	Height (cm)	LAI (cm)	Inulin content (%)	Nitrogen (N) (g kg^-1^ soil)	Phosphorus (P) (g kg^-1^ soil)	Potassium (K) (g kg^-1^ soil)
**2016**							
Control	43.83	61.033b	1.178 b	20.83 b	11.65	1.94	8.39 c
AMF	46.20	65.533b	1.565 a	41.46 a	14.06	2.13	14.71 a
PSB	43.13	63.563b	1.603 a	27.02 b	12.47	3.21	6.15 d
AMF + PSB	45.80	76.625 a	1.803 a	42.02 a	11.04	2.37	11.61 b
Grand mean	44.74	66.69	1.54	32.83	12.31	2.41	10.22
F-test	ns	*	*	**	ns	ns	**
% CV	5.82	10.24	14.86	16.74	13.61	24.61	12.14
**2017**							
Control	43.55	48.50 b	1.900 c	29.62 c	9.28 b	2.12 b	4.54 b
AMF	42.25	59.75 a	2.575 ab	31.74 b	11.36 a	2.77 b	14.00 a
PSB	40.60	53.25 b	2.675 a	33.20 a	9.78 b	3.98 a	6.25 b
AMF + PSB	43.08	65.25 a	2.275 b	28.36 d	9.66 b	2.57 b	5.12 b
Grand mean	42.37	56.69	2.36	30.73	10.02	2.86	7.48
F-test	ns	**	**	**	**	**	**
% CV	11.51	6.51	9.01	1.92	4.39	20.30	24.05

Means followed by the same letter in the same column are not significantly different according to LSD.

**Significantly different at *P* ≤ 0.01; *Significantly different at *P* ≤ 0.05; ns, not significantly different.

Table 3 showed the combined analysis of variance on SPAD values, LAI, plant height, inulin content and plant nutrient uptake (N, P, and K) of sunchoke treated by PSB and AMF in both 2016 and 2017. The results indicated that the experimental year had a significant effect on LAI, plant height and plant nutrient uptake. Likewise, the effect of treatment in both years was significant with almost all parameters, except SPAD value. When considering the interaction between the experimental year and treatment, it was found that LAI, inulin content and K uptake were significantly affected, while the other parameters were not affected. These results, which were in agreement with the results in Table 2, suggested that the experimental year, treatment and the interaction between the experimental year and treatment had an effect on the growth of sunchoke.

**Table 3:** Combined analysis on SPAD value, leaf area index (LAI), height, inulin content and plant nutrient uptake (Nitrogen, Phosphorus and Potassium) of sunchoke treated by PSB and AMF in 2016 and 2017.

	SPAD value	Height	LAI	Inulin content	Nitrogen	Phosphorus	Potassium
**Source of variation (F values)**							
Year	3.69ns	26.21**	138.33**	1.80ns	18.03**	5.14*	19.64**
Treatment	0.87ns	12.68**	16.25**	11.00**	4.07*	11.77**	37.86**
Year × Trt	0.38ns	0.57ns	3.97*	12.91**	0.33 ns	0.61ns	6.01**

Means followed by the same letter in the same column are not significantly different according to LSD.

**Significantly different at *P* ≤ 0.01; *Significantly different at *P* ≤ 0.05; ns, not significantly different.

Table 4 showed the effect of AMF and PSB inoculation on sunchoke production during 2016 and 2017. The results showed that in 2016 the effects of co-inoculation of AMF and PSB to sunchoke production were more significant than the effects of AMF or PSB alone. Plants treated with both AMF and PSB had a significant increase in tuber fresh weight (TFW), weight of individual tuber (WIT), stem dry weight (SDW), tuber dry weight (TDW) and biomass. It was found from the results in 2016 that PSB played a more important role in enhancing the production of sunchoke than AMF. However, the number of tubers (NT) obtained per plant was relatively equal in all inoculated plants, which was not significantly different from that of the uninoculated ones. In contrast, the results in 2017 clearly showed that the number NT was significantly higher in all inoculated plants than that of the uninoculated controls. Also, the effects of AMF to the production of sunchoke were significantly higher than the effects of PSB alone or even co-inoculation of both AMF and PSB. Plants treated with AMF alone had a significant increase in NT, TDW and biomass, while plants treated with both AMF and PSB had an increase in NT only. These results suggested that the experimental year had a significantly different effect on the production of sunchoke.

**Table 4:** Effect of PSB and AMF inoculation on plant growth parameters of sunchoke.

Treatments	NT	TFW (kg ha^-1^)	WIT (g)	SDW (kg ha^-1^)	LDW (kg ha^-1^)	TDW (kg ha^-1^)	Biomass (kg ha^-1^)
**2016**							
Control	14.40	7281 b	27.51 b	144.08 b	296.19 c	1743.3 b	2183.6 b
AMF	14.50	7625 b	45.41 ab	276.57 a	506.91 ab	1642.4 b	2425.9 b
PSB	10.73	9215 a	48.23 a	275.11 a	542.32 a	1470.1 b	2287.5 b
AMF + PSB	11.90	10,575 a	63.36 a	265.99 a	411.21 bc	2360.1 a	3037.3 a
Grand mean	12.88	8673.9	46.13	240.44	439.16	1804.0	2483.6
F-test	ns	**	*	**	**	*	*
% CV	20.6	10.81	25.52	18.91	18.38	17.77	15.08
**2017**							
Control	4.81 b	6836 b	29.73	469.51	617.28	1953.4 b	3040.1 b
AMF	9.13 a	9095 b	36.59	528.14	733.03	2825.5 a	4224.9 a
PSB	10.08 a	13,240 a	35.72	479.23	673.41	1984.7 b	3220.7 b
AMF + PSB	11.00 a	10,354 ab	37.77	529.71	693.87	2162.6 b	3592.1 b
Grand mean	8.75	9881.0	34.95	501.65	679.40	2231.50	3519.0
F-test	**	*	ns	ns	ns	*	**
% CV	16.49	23.40	32.80	14.00	11.37	18.04	10.96

The number of tubers per plant (NT), tuber fresh weight (TFW), weight of individual tuber (WIT), stem dry weight (SDW), leaf dry weight (LDW), tuber dry weight (TDW) and plant biomass evaluated at the harvest stage in 2016 and 2017.

Means followed by the same letter in the same column are not significantly different according to LSD.

**Significantly different at *P* ≤ 0.01; *Significantly different at *P* ≤ 0.05; ns, not significantly different.

Combined analysis of variance on sunchoke production is presented in Table 5. The results showed that the experimental year had a significant effect on all plant production parameters except TFW. The effects of each treatment on all of the plant production parameters at the harvest stage, except NT, were significantly different. The interaction of Year x Trt also had a significant effect on NT, TDW, plant biomass and harvest index. All of these results suggested that the experimental year and treatments had a significant influence on sunchoke production.

**Table 5:** Combined analysis on the number of tubers per plant (NT), tuber fresh weight (TFW), weight of individual tuber (WIT), stem dry weight (SDW), leaf dry weight (LDW), tuber dry weight (TDW) and plant biomass (Biomass) of sunchoke treated by AMF and PSB in 2016 and 2017.

Combined over two years							
	NT	TFW	WIT	SDW	LDW	TDW	Biomass
**Source of variation (F values)**							
Year	31.79**	3.76ns	8.30**	158.21**	78.61**	11.75**	63.58**
Treatment	1.89ns	9.44**	5.42**	4.53*	7.54**	4.69*	8.22**
Year × Trt	8.38**	2.75ns	2.18ns	1.45ns	2.32ns	5.44**	4.23*

Means followed by the same letter in the same column are not significantly different according to LSD.

**Significantly different at *P* ≤ 0.01; *Significantly different at *P* ≤ 0.05; ns, not significantly different.

In order to investigate the effects of PSB and AMF on plant growth and yield parameters, correlation analysis between PSB population, AMF spore density and %AMF colonization and those plant parameters in each year was carried out (Table 6). The results showed that in 2016, PSB population and AMF spore density positively correlated with almost all plant parameters, while %AMF colonization positively correlated only with LAI, P and K contents and inulin content. Contrarily, PSB population, AMF spore density and %AMF colonization positively correlated with some but not all plant parameters in 2017, i.e., plant biomass, TFW, LAI and plant height. It was found that nutrient contents and inulin content in sunchoke grown in 2017 were not significantly correlated with the presence of either PSB or AMF. However, the result clearly indicated that P content in plants was positively correlated with PSB population in both experimental years. This suggested that PSB played an important role in providing available P to plants, as was expected. Moreover, the PSB population had a significantly positive correlation with AMF spores and colonization in both years. This was evidence that PSB had synergistic effects on AMF.

**Table 6:** Correlation between the PSB population, AMF spore density, the percentage of AMF colonization, plant biomass, tuber fresh weight (TFW), leaf area index (LAI), height, nutrient uptake (N, P, K) and inulin content of sunchoke in 2016 and 2017.

Correlation	PSB population		AMF spore density		**%**AMF colonization	
	2016	2017	2016	2017	2016	2017
AMF spores	0**.**73**	0**.**81**				
AMF colonization	0**.**56*	0**.**72**	0**.**65**	0**.**83**		
Biomass	0**.**67**	0**.**45 ns	0**.**50*	0**.**65**	0**.**41 ns	0**.**62*
TFW	0**.**79**	0**.**52*	0**.**63**	0**.**32 ns	0**.**43 ns	0**.**61*
LAI	0**.**75**	0**.**73**	0**.**74**	0**.**57*	0**.**52**	0**.**67**
Height	0**.**62**	0**.**70**	0**.**67**	0**.**80**	0**.**35 ns	0**.**82**
N	0**.**47 ns	**-**0**.**03 ns	0**.**60*	0**.**24 ns	0**.**57 ns	0**.**39 ns
P	0**.**79**	0**.**52*	0**.**66**	0**.**37 ns	0**.**62*	0**.**33 ns
K	0**.**51*	0**.**041 ns	0**.**60*	0**.**32 ns	0**.**67**	0**.**34 ns
Inulin content	0**.**73**	0**.**03 ns	0**.**68**	**-**0**.**23 ns	0**.**70**	**− **0**.**22 ns

**Significantly different at *P* ≤ 0.01; *Significantly different at *P* ≤ 0.05; ns, not significantly different.

Table 7 showed combined analysis on the PSB population in rhizosphere soil, the amount of AMF spores in soil and the percentage of AMF colonized in the roots of sunchoke in 2016 and 2017. The results indicated that treatment significantly affected the PSB population, the number of AMF spores and the percentage of AMF colonization. This implied that the presence of PSB and AMF in soils was due to the inoculation of those microorganisms, but not to the indigenous soil microflora. In contrast, a significant effect of the experimental year and Year x Trt was found only on the percentage of AMF colonization. This suggested that the efficiency of AMF colonization depended on the year of experiment and the combined year and treatment.

**Table 7:** Combined analysis on the PSB population in rhizosphere soil, the amount of AMF spore in soil and the percentage of AMF colonized in sunchoke roots in 2016 and 2017.

	PSB population in rhizosphere soil	Amount of AMF spores	Percentage of AMF colonization
**Source of variation (F values)**			
Year	0.12ns	0.52ns	43.77**
Treatment	90.47**	11.45**	57.34**
Year × Trt	1.08ns	0.18ns	10.59**

Means followed by the same letter in the same column are not significantly different according to LSD.

**Significantly different at *P* ≤ 0.01; *Significantly different at *P* ≤ 0.05; ns, not significantly different.

Due to different contents of nutrients (N, P, K) between 2016 and 2017, it was worth investigating whether these nutrients were correlated with plant growth parameters or not. The correlation analysis results between nutrient contents and plant biomass, TFW, LAI, plant height and inulin content were shown in Table 8. The results clearly showed that P content was correlated with plant parameters more than N and K contents in both years. This confirmed our hypothesis that an increase in available P via an inoculation of PSB could enhance growth of sunchoke. Also, the addition of PSB in order to increase available P to plants likely helped AMF to promote plant growth. In 2017, N and K did not significantly correlate with any plant parameters, whereas in 2016 these nutrients positively correlated with all plant parameters, except LAI and inulin content. These results suggested that nutrients in 2016 had a more significant effect on sunchoke growth than those in 2017.

**Table 8:** Correlation between nutrient concentrations (N, P, K), plant biomass, tuber fresh weight (TFW), leaf area index (LAI), height, and inulin content of sunchoke in 2016 and 2017.

Correlation	Nitrogen		Phosphorus		Potassium	
	2016	2017	2016	2017	2016	2017
Biomass	0.692**	0.424ns	0.625*	0.078ns	0.654**	0.600*
TFW	0.710**	0.381ns	0.787**	0.559*	0.543*	0.238ns
LAI	0.390ns	0.418ns	0.570*	0.686**	0.439ns	0.482ns
Height	0.708**	0.311ns	0.654**	0.291ns	0.661**	0.217ns
Inulin content	0.474ns	0.323ns	0.545*	0.595*	0.756**	0.340ns

**Significantly different at *P* ≤ 0.01; *Significantly different at *P* ≤ 0.05; ns, not significantly different.

## Discussion

In our previous study, the PSB strain *Klebsiella variicola* and AMF strain *Rhizophagus intraradices* were proved to synergistically enhance sunchoke growth and its tuber quality, especially increasing inulin content in pot experiments^25^. In this work, the effects of co-inoculation of the PSB and the AMF together with single inoculations of either one of them on the growth and production of sunchoke were further investigated in field experiments. We found that there were some effects of co-inoculation of our AMF and PSB when tested in field experiments which were similar to what was found in pot experiments. Figure 1 showed that at the harvest stage, PSB populations in co-inoculated and PSB-treated plants were significantly higher than that in the uninoculated and AMF-treated plants. This is in agreement with the results from pot experiments where the PSB population was higher in co-inoculated plants than the AMF-inoculated ones^25^. This confirmed that our PSB could symbiotically live with AMF and sunchoke in natural conditions. Moreover, %root colonization by AMF was higher in co-inoculated and AMF-treated plants than in the uninoculated controls, especially in 2017, which was similar to what was observed in pot experiments. In terms of the effects to plant growth parameters, we also found that AMF and PSB synergistically enhance plant height, biomass and leaf area of sunchoke both in pot and field conditions. This suggested that our AMF and PSB were effective in promoting plant growth in both pot and field conditions. We also found that there was an increase in some plant growth parameters which were in agreement between 2 experimental years. A significant increase in P content in the PSB-treated plants in 2016 and 2017 confirmed that P solubilization activity of our PSB was effective in the fields, and thus provided available P for plant uptake. This result was similar to other reports showing that PSB could improve the growth and significant increase P of plants. This is, for example, Chen et al.^26^ reported that a significant increase in biomass and total P of wheat under both pot and field conditions were an effect of the inoculation of a *Phosphobacterium* strain. An increase in biomass of maize plants under field conditions was found in plants inoculated with *Serratia marcescens* and *Pseudomonas* sp.^27^. In addition Abel, (2011)^28^ claimed that the effects of two PSB species, *Bacillus aryabhattai* and *Pseudomonas auricularis* and a mixture of both strains showed a significant promotion on the growth of *Camellia oleifera* Abel. Remarkably, our work is the first to report a PSB strain belonging to the species *Klebsiella variicola* that could promote the growth and production of sunchoke under field condition. Interestingly, the results in our work indicated that co-inoculation of AMF and PSB could better enhance leaf area index (LAI) and plant height than the uninoculated and single-inoculated plants. These synergistic effects between AMF and PSB in promoting plant growth were also in agreement with our previous report in the pot experiments^25^. Moreover, there was a research indicating that an increase in LAI was a result of an increasing available P by the activity of PSB, thus led to an increase in plant height^29^. Therefore, the results from 2-year field experiments confirmed that our AMF and PSB had synergistic effects on increasing leaf area and plant height. This claim was also supported by a correlation analysis that LAI and plant height were significantly affected by PSB population and AMF spore density and colonization (Table 6), whereas plant nutrients (N, P, K) had less effect on them (Table 8).

Furthermore, the results in either 2016 or 2017 showed that the effects of co-inoculation of AMF and PSB outstood the other treatments in promoting plant growth and yield. In 2017, we found that the number of tubers obtained from co-inoculated plants was ~ 2 times more than that from the uninoculated plants. In 2016, stem dry weight, inulin content and K content of plants inoculated with both AMF and PSB was higher than the uninoculated controls. However, the inulin content in co-inoculated plants was relatively equal to that of the AMF-inoculated plants. Also, the highest K content was found in plants inoculated with AMF. These results were in agreement with Nacoon et al.^30^. Although it was likely that AMF played a more important role in promoting inulin and K accumulation in sunchoke, our correlation analysis (Table 6) suggested that both PSB and AMF played a significant role in enhancing these plant growth parameters. Therefore, co-inoculation of both PSB and AMF seemed to help plant growth more than a single inoculation of either of them.

Likewise, the results from our field experiments indicated that co-inoculation of AMF and PSB could better enhance plant growth than the other treatments in several ways. We found that plants inoculated with co-culture of AMF and PSB in 2016 had ~ 1.5–2 times higher in tuber fresh weight and weight of individual tubers than the uninoculated plants. Remarkably, the co-inoculation of AMF and PSB could significantly enhance tuber dry weight and plant biomass to nearly 1.5 times higher than the uninoculated controls, while plants treated with single inoculation showed no significant increase in TDW and biomass. These results clearly evidence that synergistic effects of AMF and PSB could promote yield of sunchoke under field conditions. Note that chemical and physiological properties of soils used in field experiments in 2016 and 2017 were slightly different, which were also different from those of soils used in our previous work in pot experiments. The combined analysis (Tables 3, 5) between plant growth parameters and the year of experimentation also suggested that plant growth and yield parameters were significantly affected by experimental years. Our findings still pointed out that several plant growth and yield parameters of co-inoculated plants were outstanding when compared to the uninoculated plants or even to the single-inoculated ones. These results strongly suggested that synergism of our AMF and PSB could effectively enhance growth and yield of sunchoke in a variety of field environments where changes of climates during cultivation were uncontrollable. Therefore, further investigation on the specific environmental factors in each experimental year that affect how AMF and PSB synergism promotes plant growth is worth carried out. In addition, the production of AMF and PSB inoculums for use as biofertilizers to promote growth of sunchoke under field conditions is planned for the future.

## Conclusions

This study demonstrated synergistic effects of an AMF strain *Rhizophagus intraradices* KKU-Wh and a PSB strain *Klebsiella variicola* UDJA102x89-9 to promote growth of sunchoke under field trial conditions. The results showed that the experiment year, treatment and interaction between year and treatment had an effect on the growth and production of sunchoke, and also the PSB and AMF status in soil. AMF colonization in roots and PSB population were found at the harvest stage in both experimental years, suggesting successful AMF colonization and PSB survival throughout the experiment. In 2016, the results showed that the co-inoculation of AMF and PSB (AMF + PSB) could significantly improve the growth and production of sunchoke better than an inoculation of AMF or PSB alone. In this regard, plants with co-inoculation had a significant increase in many parameters including TFW, WIT, SDW, TDW and plant biomass. In addition, plants with co-inoculation also had a significant increase in plant height, LAI and inulin content in both years. On the contrary, the results in 2017 showed that the inoculation of AMF alone played a more important role in enhancing plant growth and production than the other treatments. AMF-inoculated plants had a significant increase in NT, TDW and biomass, while plants treated with co-inoculation had only an increase in NT. Interestingly, the height of plants with co-inoculation was significantly higher than the other treatments in both years, suggesting efficient plant growth promotion due to synergistic effect of AMF and PSB. The correlation analysis showed that PSB population and amount of AMF spores had a positive correlation with all plant parameters in 2016, whereas in 2017, the correlation was found with some plant growth parameters. Moreover, PSB population in rhizosphere soil positively correlated with P content in plants, AMF spores and %AMF root colonization. This was evidence that PSB had synergistic effects on AMF development and also a cause of an increase in available P content in plants. Therefore, this work is the first to confirm successful plant growth promotion in sunchoke grown under field conditions. Note that the experimental year was also one of the statistically significant factors affecting plant growth and yield parameters. This suggested that different years of sunchoke plantation could result in different levels of plant response to the inoculation of our AMF and PSB. Further studies on environmental factors influencing the effects of synergism between AMF and PSB on plant growth promotion under field condition are worth carrying out.

## Materials and methods

### Preparation of plant seedlings

The Jerusalem artichoke seedlings (cv. HEL65) were obtained from Peanut Jerusalem Artichoke and Cassava Research Group, Agricultural Faculty, Khon Kaen University, Thailand, with permission from Assoc. Prof. Dr. Bhalang Suriharn who is head of this research group and received permission from Prof. Dr. Sanun Jogloy, this research group coordinator and co-author of this work. Plant seedlings were prepared according to the method described by Ruttanaprasert et al.^31^. Briefly, the tubers were cut into small pieces having 2–3 buds per piece. Pieces of tubers were pre-sprouted in a coconut peat medium under ambient conditions for 4–7 days. They were then transferred into germinating-plug trays containing a mixture of charred rice husks and soils as a medium. The pre-sprouting seeds were incubated for 7 days for complete sprouting. The uniform and healthy seedlings were transplanted into the pots for use in the field experiments.

### Bioinoculants selection and inoculation

An AMF strain *Rhizophagus intraradices* KKU-Wh (Accession No. LC428366) and a PSB strain *Klebsiella variicola* UDJA102x89-9 (Accession No. LC373006) used in this work were obtained from the Microbiology Laboratory, Department of Microbiology, Faculty of Science, Khon Kaen University, Thailand.

In order to prepare PSB inoculum, a single colony of PSB was subcultured onto nutrient agar (NA) and incubated at 30 °C for 24 h. Then, a loopful of bacterial biomass was transferred into 25 mL of nutrient broth (NB) and incubated with shaking at 150 rpm, 30 °C, 24 h. The culture was subsequently upscaled to a volume of 200 mL and 2 L, respectively. Quantification of bacterial concentration was carried out by plate count technique. Cell pellets were retrieved by centrifugation at 6,000 rpm, 15 min. The pellets were washed twice and resuspended in 0.85% NaCl. Ten milliliters of PSB suspension with a concentration of 10^9^ CFU mL^−1^ were poured onto the plant seedlings.

AMF inoculum was propagated by the pot culture technique^32^. Maize was used as a host plant. In order to surface sterilize, maize seeds were soaked in 10% sodium hypochlorite for 30 min. AMF spores were inoculated onto the surface-sterilized maize seeds which were then sown into plastic pots containing twice-sterilized soil. Maize was grown in a greenhouse without any fertilizers or chemicals applied. After 3 months of transplantation, the maize was cut, and the soil was left to dry in the pots before use as an inoculum. Quantification of AMF spores in soil inoculum was carried out by sucrose centrifugation method^33^. The 200 g of soil inoculum containing a starting AMF concentration of 10 spores g^−1^ soil was applied to each experimental plot to a depth of 5 cm. Then, a single plant seedling was transferred into a hole where AMF inoculum was already placed at the bottom. In the case of co-inoculum treatment, 10 ml of PSB inoculum was added onto the seedlings adjacent to the roots.

### Experimental design

The field experiments were set up at an agronomy farm at Khon Kaen University (16° 28′ N, 102° 48′ E, 200 m above mean sea level). A Randomized Complete Block Design (RCBD) with four replications was carried out in a plot size of 400 m^2^ (20 × 20 m). The study was performed in duplicate for each individual experiment in 2016 and 2017. The four treatments included: control plants without any inoculation (T1); plants with a single inoculation of AMF (*Rhizophagus intraradices* KKU-Wh) (T2); plants with a single inoculation of PSB (*Klebsiella variicola* UDJA102x89-9) (T3); and plants with a co-inoculation of PSB and AMF (T4). Water was supplied to plants for 30 min once a day through a mini sprinkler system to maintain high humidity conditions. Manual weeding was carried out every 4 weeks after transplanting (DAT). Pests and diseases were not controlled until harvest.

Chemical and physical properties of soils were determined as follows: soil pH was determined with a pH meter in a suspension of 1:1 (w/v) dried soil: water. Total organic matter was measured by wet oxidation method according to Walkley and Black^34^. Total nitrogen was extracted by the Kjeldahl nitrogen method, followed by measurement using the flow injection analyzer; FIA method^35^. Available P in soil was determined by Bray-II according to Bray and Kurtz^36^. In order to determine the exchangeable K content, exchangeable cations in soils were extracted using 1 N ammonium acetate. The measurement was carried out using a flame photometer at 768 nm^37^.

### Plant growth parameter determinations

At 60 days after transplantation, plant growth parameters including SPAD value, plant height, and leaf area index (LAI) were determined. SPAD values were measured using a SPAD 502-plus (Konica Minolta, Japan). LAI was measured using an LAI-2200C plant canopy analyzer meter (LI-COR, USA). At the harvest stage (100 DAT), 12 plants per treatment of each plot were collected. Then, a total dry biomass of the plant roots, stem, and leaves were determined after drying at 80 °C for 3 days. The number of tubers per plant, tuber fresh weight and tuber dry weight were also determined. Inulin accumulation was also determined following the method described by Saengkanuk et al.^38^.

### Quantification of nitrogen, phosphorus and potassium contents

Nutrient concentrations (nitrogen (N), phosphorus (P), potassium (K)) were measured from the dried stems and leaves. Total N content was extracted from plant tissue by micro-Kjeldahl method of Bremner^39^. Thereafter, the N content was analyzed using a colorimetric method by Auto-Analyzer 3 (AA3), SEAL Analytical, Germany; Method No. G-253-00 Rev.1 (Multitest MT7/MT8) at an absorbance of 660 nm. Phosphorus was extracted using a Wet oxidation method by mixing with nitric acid and perchloric acid. Total P content was determined by a spectrophotometer at a wavelength of 420 nm by the molybdovanadate with acid persulfate digestion method^40^. Potassium was extracted from plant tissues using a Wet oxidation method by mixing with nitric acid and perchloric acid (2:1 v/v). Then, K content in the solution was detected by a flame photometer at 768 nm (Flame photometer, Model 410 Sherwood, United Kingdom)^41^.

### Determination of AMF root colonization

The percentage of plant root colonization was determined from 4 plants in each experimental plot. Staining of plant roots was carried out following a modified method described by Koske and Gemma^42^. Fresh roots were washed with tap water and sieved to remove soils. Roots were decolorized by soaking in 2.5% KOH solution for 10 min at 90 °C. Roots were then stained using 0.05% lactic acid-glycerol-Trypan blue. In order to visualize under a microscope, root samples were cut into 0.5–1.0 cm pieces and placed on glass slides. AMF structures and their root colonization were observed using a light microscope (Nikon Eclipse50i, Japan) at 40–100X magnification. The percentage of root colonization by AMF was estimated by the method described by Trouvelot et al.^43^.

### Quantification of AMF spores and PSB population

A number of AMF spores extracted from soil samples were quantified following the method described by Daniels and Skipper^33^. Briefly, 5 g of soil samples were mixed with 30 mL of tap water. Soil mixture was centrifuged at 5000 rpm for 5 min and removed supernatant. Then, 30 mL of sucrose solution (50% (w/v)) was mixed with the soil pellet. The mixture was centrifuged at 3000 rpm for 1 min to remove the AMF spores from the soil. The supernatant was filtered through a filter paper Whatman No.1 and placed on a petri dish for spore counting under a stereomicroscope (Nikon SMZ745T). The enumeration of PSB in the rhizosphere soil sample was determined by the dilution plate count technique on Pikovskaya’s agar medium. Each sample was carried out in triplicate. Plates were incubated at 30 °C for 3 days. A clear halo zone around colonies indicative of P solubilization were counted^44^.

### Statistical analysis

Data were analyzed according to a factorial in a Randomized Complete Block Design (RCBD) from four plot replications. Analysis of variance (ANOVA) was performed for plant growth and production data in each year. The two experimental years were tested by combined analysis of variance. Multiple comparisons test of the differences among the treatment means was conducted using the least significant difference (LSD) when the main effect was *P* < 0.05. Correlations among plant growth parameters were analyzed using the Pearson’s correlation coefficient and evaluated at a significant level of *P* < 0.05. All statistical analyses were performed using a Statistix 10.0 software.
